# The self-reference effect as a behavioral indicator of identity disturbances associated with borderline personality features in a non-clinical sample

**DOI:** 10.1186/s40479-022-00189-7

**Published:** 2022-07-20

**Authors:** Joseph Maffly-Kipp, Morgan N. McCredie, Leslie C. Morey

**Affiliations:** Department of Psychological and Brain Sciences, Texas A&M University, 4235 TAMU, College Station, TX 77843 USA

**Keywords:** Identity disturbance, Borderline personality disorder, Memory, Personality pathology

## Abstract

**Background:**

Identity disturbances are a common feature of personality pathology and BPD. The Self-Reference Effect paradigm is a method used to measure the impact of self-relevant processing on encoding/memory, whereby self-relevant information is typically advantaged in cognitive processes. We postulated that difficulties with identity might impede the process by which one encodes self-relevant information. Based on this reasoning, we predicted that high levels of identity disturbance could be associated with atypical impact of the SRE.

**Methods:**

Undergraduate participants were randomized into one of three groups where they were exposed to 60 trait adjectives for seven seconds each. Depending on condition, participants either indicated whether a word was/wasn’t capitalized (Capitalization condition), whether it was a good synonym for “openness” (Synonyms condition), or whether it described them as a person (Self-reference condition). After a brief delay, all participants were asked to recall as many of the 60 words as possible. Finally, we measured identity disturbance using the Borderline Features–Identity Problems (BOR-I) scale from the Personality Assessment Inventory.

**Results:**

We found significant but modest correlations between Recall and scores on the BOR-I subscale in the Self-Reference condition, but not the two control conditions. Contrary to expectations, the interaction between BOR-I and Condition was not a significant predictor of Recall, suggesting that identity disturbance did not significantly moderate the SRE.

**Conclusions:**

While our primary hypothesis was not supported, there is a need for multimethod approaches to studying personality pathology. Future research should continue to examine the extent to which behavioral paradigms like the SRE might be useful indicators of identity disturbance/personality pathology, with an emphasis on the use of clinical populations.

**Supplementary Information:**

The online version contains supplementary material available at 10.1186/s40479-022-00189-7.

## Background

Identity disturbances are persistent, atypical experiences of self that are common across a variety of psychiatric disorders [32]. They represent a core feature of borderline personality disorder (BPD), as demonstrated in its diagnostic criteria [1] and via theoretical models [19], empirical evaluation, including network analysis [35, 44], factor analysis [16], and assessment of longitudinal symptom endurance [27]. Identity problems have been linked to a variety of problematic outcomes, including substance misuse [34, 46, 47], suicidality [8, 53], and other-directed violence [13]. However, despite compelling evidence to suggest that identity disturbance is both central to BPD and of clinical relevance, the construct remains understudied relative to other BPD features [18]. Further, due to the limitations of self-report data, there is value in employing multimethod approaches for studying personality pathology in order to gain a more comprehensive view of how such pathology impacts functioning. In the present work, we investigate the relevance of a common behavioral memory paradigm, the Self Reference Effect, to identity disturbances associated with BPD.

### Identity disturbance

The current *Diagnostic and Statistical Manual of Mental Disorders* (*DSM-5*; [1]) defines identity disturbance as “markedly and persistently unstable self-image or sense of self,” as demonstrated by “sudden and dramatic shifts in self-image, characterized by shifting goals, values, and vocational aspirations” ([1], p. 664). Per the *DSM-5*’s categorical approach to personality disorder (PD) diagnosis, identity disturbance represents one of nine criteria used to diagnose BPD [1]. Kernberg [19] first theorized that identity problems were a core feature of BPD, and studies of both adults and adolescents have since corroborated this assertion [5, 33, 49]. Furthermore, the single criterion of identity disturbance has been shown to be the strongest predictor of receiving any PD diagnosis [30]. In light this “core dysfunction” view of PDs, recent advancements in dimensional PD classification (e.g., the Alternative Model for Personality Disorders, [1]) have identified core impairments in self (i.e., identity and self-direction) and interpersonal (i.e., intimacy and empathy) functioning, which are thought to represent the fundamental nature of personality pathology underlying all categorical PD diagnoses (see Sharp and Wall [40] for a detailed review). Within this conceptualization, identity problems thus assume a more central position in the diagnosis of PD broadly, and BPD can be viewed as a proxy for general impairment in personality functioning [41, 52].

### Borderline personality disorder features

Even within a dimensionalized system however, it should be recognized that the construct of borderline pathology encompasses a diverse array of other cognitive, affective, and behavioral features. One of the many long-standing criticisms of the current categorical diagnostic model is the clinical heterogeneity within PD diagnostic categories, such that individuals with the same PD diagnosis can demonstrate wide variation in symptom presentation [9, 48]. For instance, the use of poorly justified diagnostic thresholds for assigning diagnoses in the categorical model [4] requires that an individual meet five of nine diagnostic criteria in order to be diagnosed with BPD, resulting in 256 possible combinations of diagnostic criteria [14]. This poses a challenge for clinical assessment, treatment development, professional communication, and etiological research. In response, researchers have used a variety of analytic approaches in attempts to identify subtypes or underlying core dimensions of the BPD phenotype (e.g., [10, 11, 23, 37, 42, 43, 52]). For example, factor analytic studies have tended to identify three underlying dimensions of BPD: identity and relationship disturbance, affective disturbance/emotional dysregulation, and behavioral dysregulation/impulsivity [10, 37, 39]. Elevations in any single one of these factors might have meaningful implications for cognition and behavior, without an individual necessarily meeting diagnostic criteria for BPD. However, many measures of BPD symptoms are self-report in nature, which carry inherent limitations involving a person’s ability to introspect and accurately report on their own tendencies across time [7]. Thus, alternative strategies for assessing personality pathology and identity problems, behavioral or otherwise, could potentially complement the shortcomings of commonly used self-report scales.

### Self-reference effect

The self-reference effect (SRE) manipulation is a well-researched paradigm that is broadly intended to measure the role of the self in memory [20]. In 1977, Rogers and colleagues found that participants recalled significantly more trait-related adjectives when prompted for self-reference (i.e., “Does this trait describe you?”) compared to semantic (“Is this a synonym for X?”), or structural (i.e., “Is this word capitalized?”) encoding strategies [36]. They interpreted their findings to suggest that the processing of adjectives in relation to oneself increased encoding and future recall. In the decades that followed, this paradigm was used frequently in empirical investigations, often to explore how individuals process and encode self-relevant information (see [45]). While many of these studies have examined group averages in normative populations, some evidence of individual differences exists as well. For example, altruism [31], older age [12], and membership to a non-Western culture [54] have been associated with a diminished SRE. Thus, while the SRE is certainly useful for understanding the general relationship between memory and self-relevant processes, it also has the apparent potential to highlight differences in the experience of memory and self between individuals.

### SRE and psychopathology

Given the importance of identity and self to the understanding of various psychiatric disorders [21, 22], the SRE may provide useful information about psychopathology. Multiple investigations have explored this broad notion. For example, at least three studies [6, 15, 25] have found evidence of a diminished self-reference effect in individuals with Autism Spectrum Disorder (formerly diagnosed as “Asperger Sydrome”), though this finding has been disputed [24]. Separately, Jones and Brunell [17] found that narcissism was positively related to the SRE, especially for positive agentic traits. Both of these disorders are characterized by atypical self-representation. The SRE can thus help researchers to better understand the ways that self-referential processes function in various mental disorders, and can potentially serve as a behavioral indicator that complements self-report assessments. This may be particularly relevant for borderline pathology (and personality pathology more broadly), in which identity disturbances are thought to be a defining feature [29]. Previous research involving BPD and memory/processing might provide some indication of this. For example, previous investigations have found that BPD symptoms relate to a negativity bias involving positive self-relevant words [51], and better recall involving self-relevant social events [50]. Though neither of these studies investigated recall overall, or the specific impact of identity disturbance, they broadly support the notion that borderline personality pathology may lead to atypical self-referential processing. Given the conceptual importance of identity to self-relevant processing, it stands to reason that identity disturbance specifically could relate to overall differences in self-relevant memory and encoding, regardless of the valence of the self-relevant words. Though no study to our knowledge has investigated this question, doing so could aid the current understanding of how BPD features impact memory, encoding, and identity.

### The current study

Drawing on the above rationale, we examined the SRE in relation to BPD features in a nonclinical sample. Identity disturbances are marked by persistent disruptions in the experience of self, and are a core component of personality disorders, particularly BPD [1]. The SRE is a cognitive psychological paradigm that is intended to capture the impact of self-referent processing on encoding and recall [45]. Potential individual differences in the SRE based on BPD symptoms might serve as a useful starting point in the effort to create multimethod assessment strategies for identity disturbance and personality pathologies. Based on the logic that identity disturbances may impact self-referential encoding to some extent, we predicted that the SRE would differ as a function of identity problems. However, our theorizing and review of the literature led us to two opposing predictions: First since disturbances of self are a core feature of BPD, it is plausible that these disturbances may interfere with the self-relevant processing thought to drive the SRE (i.e., an individual might struggle to know whether a word describes them), thus yielding a recall deficit for individuals with a greater degree of identity impairment. However, it is also plausible that, due to struggles with self-referent processing, individuals with identity disturbances might spend more time and expend more cognitive effort in considering whether a word describes them, and thus display enhanced recall. As a result of these two lines of theorizing, we decided upon the non-directional prediction that BPD features would moderate the magnitude of the SRE. In order to test this hypothesis, we first exposed participants to the SRE paradigm, then measured BPD symptomatology. We then examined identity disturbance as a moderator of the SRE.

## Method

### Participants

Complete data were provided by a total sample of 609 (53.9% female, 45.6% male, 0.5% nonbinary/nonidentifying) undergraduate participants ranging from 18–27 years old (*M*_*age*_ = 19.17*, SD* = 1.08). Participants were recruited from a participant pool of university students enrolled in an introductory psychology course and received course credit for their participation. No exclusion criteria were imposed during recruitment. The majority of these participants identified as White/Caucasian (80.0%), with the remainder of participants identifying as Asian (13.7%), Black/African-American (4.0%), American Indian/Alaska Native (2.0%), and Native Hawaiian/Pacific Islander (0.3%). A total of 27.3% identified as being of Hispanic or Latino ethnicity. Twenty-five (4.1%) participants obtained raw scores of 9 or above on the Personality Assessment Inventory (PAI) Infrequency scale [28], an indicator designed to detect careless or random responding,as such, these participants were excluded from analyses due to concerns regarding invalid responding. A further 56 participants were excluded for failing the integrity check questions [3]. Thus, our final sample consisted of 528 participants.

### Procedure

Participants signed up for this study on a voluntary basis through the participant pool website at Texas A&M. The use of mobile devices was prohibited. They were then directed to an information page where they were informed of their rights as participants, reminded that their participation was voluntary, and provided with contact information for the local IRB that approved this research. They then completed all study materials through the online survey software Qualtrics using personal computers. After completing all study materials, they read a debriefing page which informed them of the true aims of the study, and their participation was completed.

### Materials

#### Self-reference paradigm

We chose to administer this paradigm in a between-participants manner based on meta-analytic research demonstrating that this approach yields similar effect sizes, and eliminates the potential for condition interference, compared to a within-subjects design [45]. Sixty words from the Introversion/Extraversion and Openness/Intellect domains of the Big-5 were selected for inclusion in this paradigm, as previous investigations have not indicated reliable differences between individuals with BPD and the general population on these domains [38]. First, participants were randomly assigned to one of three conditions for word presentation, in which they were given seven seconds to view a single word on a screen and answer “True” or “False” before the screen automatically advanced to the next word. In the “Capitalization” condition, participants were simply asked to identify whether the word presented was capitalized (half of the words were, half were not). In the “Synonyms” condition, participants were asked whether they considered the word as a possible synonym for “openness”. In the “Self-reference” condition, participants were asked whether each word described them as a person. The use of two different control conditions is common in SRE paradigms, in order to rule out the possibility that differences between control and experimental conditions are the result of simple depth of processing [45]. Therefore, the capitalization condition represents simple orthographic engagement, while the synonyms condition involves semantic engagement, and the self-reference condition involves semantic engagement as well as self-relevant processing. In all conditions, the word and the two response choices were the only stimuli on the screen, along with the generic prompt that stayed the same for all words (e.g., “is this word capitalized?”). After being exposed to all 60 words, participants from all conditions answered five simple math questions, which served as a delay task. Finally, all participants read the instructions: “In the first task of this experiment, you responded ‘yes’ or ‘no’ to a list of 60 words. In the space below, please write as many of those words as you can remember”, and were given 5 min to type as many words as they could recall into a blank box. Using a free recall outcome variable is quite common in research that uses the SRE paradigm, and typically yields similar results to a recognition task [2]. The number of correct words that participants typed (regardless of incorrect words) was scored as our primary dependent variable: Recall.

#### Personality Assessment Inventory‒Borderline Features (PAI-BOR)

The PAI-BOR scale [28] consists of 24 self-report items which assess the primary symptoms associated with a diagnosis of BPD. This scale utilizes a four-point response format (ranging from *False, Not at all True* to *Very True* and includes four subscales: Affective Instability (BOR-A, Identity Problems (BOR-I, Negative Relationships (BOR-N, and Self-Harm (BOR-S; impulsivity. PAI scale and subscale raw scores are linearly transformed to T-scores (mean of 50, standard deviation of 10 to provide interpretation relative to a standardization sample of 1,000 community-dwelling adults that was selected to match U.S. census projections on the basis of gender, race, and age. As such, the PAI offers a useful, brief dimensional assessment of BPD features among nonclinical respondents relative to the general population. Furthermore, the BOR scale correlates with the Levels of Personality Functioning Scale–Self-Report, a measure of core personality impairment, very highly (in the range of 0.75-0.80; see [29]. The PAI-BOR scale demonstrated high internal consistency (α = 0.87) in the present sample. Descriptive and scale statistics for all PAI-BOR subscales are presented in Table 1. Our primary analyses focus on the assessment of identity disturbance using the BOR-I subscale; notably, a full 83.4% of our sample was above the community mean on BOR-I, and 25.7% were above the cut-off indicating clinical significance (70* T*).

**Table 1:** Descriptive statistics and correlations between primary study variables

	*M*	*SD*	Range	*α*	1.	2.	3.	4.	5.	6.
1. BOR	61.7	10.89	35–94	.87	-					
2. BOR-I	62.7	10.97	36–89	.68	.839^**^	^−^				
3. BOR-A	58.5	11.37	36–91	.77	.835^**^	.584^**^	-			
4. BOR-N	59.1	10.50	34–87	.65	.797^**^	.611^**^	.571^**^	-		
5. BOR-S	56.1	11.82	37–99	.69	.660^**^	.406^**^	.424^**^	.313^**^	-	
6. Words Recalled	9.66	6.70	0–31	-	.079^*^	.104^**^	.081^*^	.039	-.012	-

BOR scores are presented as *T*-scores with a mean of 50 and standard deviation of 10. ^*^p < .05; ^**^p < .01

#### Personality Assessment Inventory‒Infrequency (PAI-INF)

The PAI-INF scale [28] consists of 8 self-report items with very low endorsement rates among both normative and clinical samples, thus indicating a pattern of infrequent responses that is uncorrelated with psychopathology. Raw scores of 9 (75* T*) or above on PAI-INF have been recommended as a cut score for identifying careless or random responding [28]. The PAI-INF was used in the current study in addition to integrity check questions [3] to exclude participants on the basis of concerns regarding invalid responding.

### Data analysis

All analyses were conducted using SPSS (Version 26; IBM Corp, Armonk, NY). Our planned analyses included the calculation of bivariate correlations between primary study variables, a between-groups Analysis of Variance (ANOVA) and planned contrasts tests to test for the basic SRE, and a hierarchical regression to test for an interaction between identity disturbance (BOR-I) and Condition to predict word recall. We based this decision on previous literature (see Long and Freese [26]; pg. 418) suggesting that regression analyses are the most optimal way to test for an interaction between a continuous (BOR-I) and a categorical predictor. Our condition variable was dummy-coded with Self-Reference serving as the reference group (Dummy 1: Synonyms/Self-Reference = 0, Capitalization = 1; Dummy 2: Capitalization/Self-Reference = 0, Synonyms = 1). BOR-I (mean-centered) and the two dummy variables were entered into step 1 of the regression, the interaction terms (BOR-I x Dummy1; BOR-I x Dummy 2) were entered in step 2, and recall was entered as the dependent variable.

## Results

First, we conducted a between-groups ANOVA to determine whether Recall differed overall between the three experimental groups. Following from the extensive literature on the SRE (see [45]), we anticipated that there would be a significant impact of Condition on Recall, such that participants in the Capitalization condition would recall significantly fewer words than the other two conditions, and participants in the Synonyms condition would recall significantly words that those in the Self-Reference condition. As expected, there was a significant effect of Condition on Recall, *F*(2,521) = 192.39, *p* < 0.001, *η*_*p*_^*2*^ = 0.425. Further, planned contrasts tests revealed that participants in the Capitalization condition (*M* = 3.90, *SD* = 3.89) recalled significantly fewer correct words than participants in the other two conditions, *t*(521) = -17.11, *p* < 0.001, and that participants in the Synonyms condition (*M* = 9.63, *SD* = 4.96) recalled significantly fewer correct words compared to participants in the Self-Reference condition (*M* = 14.70, *SD* = 6.05), *t*(521) = -9.50, *p* < 0.001. These results confirmed that we replicated the basic self-reference effect.

We then looked at how recall related to the PAI-BOR scales. Table 1 presents the scale means and standard deviations, as well as correlations among primary study variables. Notably, we observed weak but significant correlations between Recall and the full BOR scale, as well as the Identity Problems (BOR-I) and Affective Instability (BOR-A) subscales. We then applied a Bonferroni correction based on our five correlations of interest (the relationship between Recall and each BOR scale), and determined that only the relationship between BOR-I and Recall, *r*(521) = 0.104, *p* = 0.008, met the new threshold (*p* < 0.01) for statistical significance. We unpacked that correlation by looking at the correlation between BOR-I and Recall in each of our three experimental conditions. We found a significant relationship between BOR-I and Recall in the Self-Reference condition, *r*(182) = 0.173, *p* = 0.019, but not in the Capitalization condition, *r*(158) = 0.134, *p* = 0.092, or the Synonyms condition, *r*(177) = -0.013, *p* = 0.859.

**Fig. 1:**
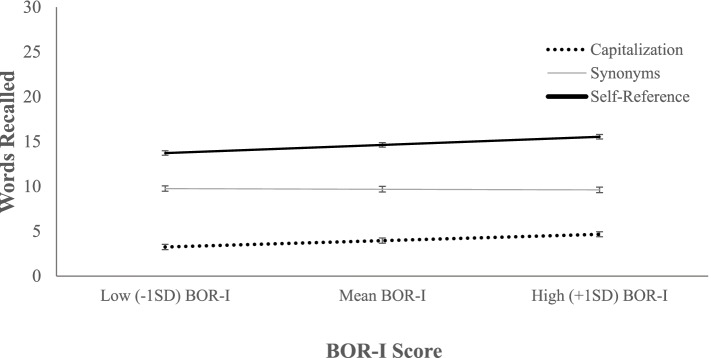
Condition x BOR-I Interaction Predicting Words Recalled. *Note*. Number of words recalled in each condition (Capitalization, Synonyms, Self-Reference) at the mean, low (-1 SD), and high (+ 1 SD) BOR-I levels is depicted. The Condition x BOR-I interaction was not significant (*p* = .143) in the moderation analysis

We next conducted a moderation analysis to determine whether the PAI BOR-I subscale interacted with the SRE to predict Recall. The main effects in step 1 accounted for a significant amount of variance, Δ*R*^2^ = 0.431, *F*(3, 519) = 131.01, *p* < 0.001. The addition of the interaction terms in step 2 revealed that the Condition x BOR-I interaction was not significant, Δ*R*^2^ = 0.004, *F*(2, 517) = 1.95, *p* = 0.143 (see Fig. 1). The BOR-I x Dummy 1 interaction was not significant, *b* = -0.053, *SE* = 0.16, *t*(522) = 0.34, *p* = 0.736, 95%CI [-0.37, 0.26], and there was a marginal interaction between BOR-I and Dummy 2, *b* = -0.265, *SE* = 0.14, *t*(522) = 1.86, *p* = 0.064, 95%CI [-0.54, 0.02]. There was, however, a significant main effect of BOR-I, *b* = 0.249, *SE* = 0.11, *t*(521) = 2.35, *p* = 0.019, 95%CI [0.04, 0.46]. While we did not probe the interaction because it was nonsignificant, the main effect of BOR-I suggests that there was a significant effect of BOR-I on recall in the reference group (self-reference condition) in this model.

## Discussion

Given the limitations of self-report, multimethod approaches to assessing personality pathology are needed, including behavioral assessments. In the present study we conducted an exploratory investigation of the SRE paradigm as a behavioral method to evaluate identity disturbance associated with personality pathology and borderline pathology specifically. Scores on all BOR scales were also well above the means of the PAI normative sample, supporting the utility of studying these questions in an undergraduate sample as a preliminary investigation. Our results offered considerable support for the SRE paradigm, such that the total number of words correctly recalled was significantly higher in the self-reference condition as compared to the capitalization and synonym conditions. Contrary to expectations, however, the relationship between identity disturbance and words recalled was not significantly different between experimental conditions, although there was some indication of a trend towards individuals with identity problems recalling more self-relevant words. 

There are a variety of possible explanations for these null findings. One possible explanation is that identity disturbances minimally/inconsistently relate to greater levels of processing/encoding of self-relevant adjectives. Indeed, there is some precedence for this finding with respect to other clinical disorders for which sense of self plays a key role; for instance, some studies have found that the self-reference effect is undiminished in individuals with Autism Spectrum Disorder despite the atypical self-awareness associated with the disorder (e.g., [24]). It is also possible that the SRE is not a particularly sensitive test of this phenomenon. For example, it is plausible that identity disturbances do relate to small differences in self-relevant encoding, however, as suggested by the proportion of variance accounted for by the main effects, the strength of the SRE made the detection of moderating variables extremely difficult. Future investigations could attempt to determine whether different variations on the SRE paradigm might be more sensitive tests, for example those that utilize within-subjects designs or differentiate between negative and positive self-referential adjectives (see [45] for other variations).

We would like to cautiously note that we did observe nonsignificant trends for the moderating role of the BOR-I scale, and there was a significantly positive, but modest, relationship between BOR-I and Recall in the self-reference condition. We observed this pattern at the level of bivariate correlation, as well as in our regression model. We acknowledge that we are limited in our capacity to interpret the significance of this main effect in light of the non-significant interaction; however, we do believe that this finding points to the need for further research regarding the effect of identity impairment on basic cognitive processes such as memory and encoding. This finding has some consistency with previous work suggesting that people with BPD pathology demonstrate more specific enhancements in memory of self-relevant social events [50]. The possibility that identity issues specifically might relate to an *enhanced* encoding process in the context of self-relevant information could reflect a similar/processing attentional bias towards information that is relevant to one’s symptomatology. This topic clearly deserves further investigation, particularly considering the central role of identity disturbance in contemporary PD classification models.

This work has several important limitations. First, the study relied on self-report measures in an online setting, which could have impacted engagement and data quality. Second, given the exploratory nature of this research, we relied on a non-clinical population, which was composed of predominantly young, white undergraduates. Although the use of a non-clinical sample hinders our ability to make direct inferences about BPD as a clinical construct, it was our intention that this study would serve as a preliminary investigation that would ultimately guide future research in clinical populations. Furthermore, the fact that we observed high means across the BPD scales, and a that full 25.7% of our sample was above the clinical cutoff for BOR-I, might suggest that this investigation has some clinical relevance. Finally, the design only involved recall assessment, and the SRE has been studied using a myriad of different conditions and outcome variables. All of these factors limit the extent to which our results can be confidently interpreted as informing theoretical conceptualizations of psychopathology, especially in regards to causal inference. Future work should approach this research question by attempting to determine causality in more controlled experimental settings, with an emphasis on the use of clinical populations. Despite these limitations, this investigation represents an important cross-disciplinary step towards understanding self-relevant processing and recall in the context of identity disturbances.

## Conclusions

The present work investigated the utility of a common memory/encoding paradigm, the self-reference effect, in exploring and predicting identity disturbances related to borderline personality pathology. While words that were encoded in relation to the self were recalled significantly more overall compared to both comparison conditions, this difference did not meaningfully differ based on individual differences in identity disturbance. Future work should continue to investigate nontraditional approaches to measuring personality pathology and self-concept in order to work towards more comprehensive multimethod assessment.

## Supplementary Information

**Additional file 1.** Supplemental Analyses.

## Data Availability

Our data are available upon request.
