# Supplementary material for: The self-reference effect as a behavioral indicator of identity disturbances associated with borderline personality features in a non-clinical sample
Source: Borderline Personal Disord Emot Dysregul. 2022 Jul 20;9:20. doi: 10.1186/s40479-022-00189-7 (PMC9297558; doi:10.1186/s40479-022-00189-7)
Supplement: Supplementary file 1 — Additional file 1. Supplemental Analyses. [file 40479_2022_189_MOESM1_ESM.docx]

**Supplemental Analyses**

First, we tested whether the full BOR scale would interact with Condition to predict Recall. There was no evidence of a Condition x BOR interaction, Δ*R*^2^ = .001, *F* (2, 518) = 0.45, *p* = .640. Next, we tested for an interaction between the BOR-I subscale and Condition. Similarly, we found no evidence of a Condition x BOR-I interaction, Δ*R*^2^ = .004, *F* (2, 517) = 1.95, *p* = .143. There was also no evidence of a two-way interaction between Condition and the BOR-A subscale, Δ*R*^2^ = .003, *F* (2, 518) = 1.12, *p* = .328, or the BOR Negative Relationships (BOR-N) subscale, Δ*R*^2^ < .001, *F* (2, 517) = 0.93, *p* = .930, to predict Recall.

Finally, we examined whether the Self-Harm/Impulsivity (BOR-S) subscale would interact with Condition to predict Recall. The dummy coded condition variables and the BOR-S scale were entered into step one of a linear regression, and the two-way interaction terms were entered into step 2. The results revealed that the main effects accounted for a significant amount of variance, Δ*R*^2^ = .436, *F* (3, 518) = 133.68, *p* < .001. However, this was qualified by a significant BOR-S x Condition interaction, Δ*R*^2^ = .007, *F* (2, 516) = 3.21, *p* = .041. To probe this interaction, we looked at the relationship between BOR-S and Recall within each condition. BOR-S did not significantly predict Recall in the Capitalization condition, *b* = .156, SE = .13, *t*(521) = 1.16, *p* = .248, 95%CI [-.11, .42], or in the Synonyms condition, *b* = -.187, SE = .12, *t*(521) = -1.54, *p* = .124, 95%CI [-.42, .05]. There was, however, a significantly negative relationship between BOR-S and Recall in the Self-Reference condition, *b* = -.288, SE = .12, *t*(521) = -2.38, *p* = .018, 95%CI [-.53, -.05].

**Exploratory Analyses**

The moderation analyses for the BOR-I subscale showed a nonsignificant trend in the direction of an increased SRE for those higher in BOR-I. As an exploratory step, we probed the nonsignificant interaction, and found that, while the relationship between BOR-I and words recalled was not significant in the Capitalization, *b* = .195, SE = .12, *t*(521) = 1.65, *p* = .099, 95%CI [-.04, .43], or Synonyms, *b* = -.017, SE = .10, *t*(521) = -0.17, *p* = .861, 95%CI [-.20, .17], conditions, it was significant in the Self-Reference condition, *b* = .249, SE = .11, *t*(521) = 2.35, *p* = .019, 95%CI [.04, .46]. The significant moderation effects found for the BOR-S subscale, however, yielded a significant pattern in the opposite direction. We therefore thought it was possible that some trait(s) captured by the BOR-S scale (e.g., impulsivity) was suppressing a potential interaction with the BOR-I scale. To explore this possibility, we computed a difference score by subtracting BOR-S scores from BOR-I scores.

We ran a regression by entering the same dummy coded condition variables and the BOR Difference scale into Step 1, and the two-way interaction terms into Step 2. The main effects accounted for a significant amount of variance, Δ*R*^2^ = .445, *F* (3, 518) = 138.25, *p* < .001. As expected, this was qualified by a significant interaction (see Figure 1) between BOR Difference and Condition to predict Recall, Δ*R*^2^ < .007, *F* (2, 516) = 3.15, *p* = .044. To probe this interaction, we looked at the relationship between BOR Difference and Recall within each Condition. There was no significant association in the Capitalization condition, *b* = .076, SE = .12, *t*(521) = 0.66, *p* = .512, 95%CI [-.15, .30], or the Synonyms condition, *b* = .092, SE = .09, *t*(521) = 0.98, *p* = .330, 95%CI [-.09, .28]. However, BOR Difference positively predicted Recall in the Self-Reference Condition, *b* = .396, SE = .10, *t*(521) = 3.96, *p* < .001, 95%CI [.20, .59].
